# Forward-Looking Ultrasound Wearable Scanner System for Estimation of Urinary Bladder Volume

**DOI:** 10.3390/s21165445

**Published:** 2021-08-12

**Authors:** Hyeong Geun Jo, Beom Hoon Park, Do Yeong Joung, Jung Ki Jo, Jeong-Kyu Hoh, Won Young Choi, Kwan Kyu Park

**Affiliations:** 1 Department of Convergence Mechanical Engineering, Hanyang University, Seoul 04763, Korea; sjdf5702@hanyang.ac.kr (H.G.J.); pbh128@hanyang.ac.kr (B.H.P.); naver2do@hanyang.ac.kr (D.Y.J.); 2 Department of Urology, College of Medicine, Hanyang University, Seoul 04763, Korea; 3 Department of Obstetrics and Gynecology, College of Medicine, Hanyang University, Seoul 04763, Korea; hohjk@hanyang.ac.kr; 4 Division of Intelligent Robotics, Daegu Gyeongbuk Institute of Science and Technology (DGIST), Daegu 42988, Korea; choiwy@dgist.ac.kr

**Keywords:** bladder volume, least-squares method, ultrasound, wearable device

## Abstract

Accurate measurement of bladder volume is an important tool for evaluating bladder function. In this study, we propose a wearable bladder scanner system that can continuously measure bladder volume in daily life for urinary patients who need urodynamic studies. The system consisted of a 2-D array, which included integrated forward-looking piezoelectric transducers with thin substrates. This study aims to estimate the volume of the bladder using a small number of piezoelectric transducers. A least-squares method was implemented to optimize an ellipsoid in a quadratic surface equation for bladder volume estimation. Ex-vivo experiments of a pig bladder were conducted to validate the proposed system. This work presents the potential of the approach for wearable bladder monitoring, which has similar measurement accuracy compared to the commercial bladder imaging system. The wearable bladder scanner can be improved further as electronic voiding diaries by adding a few more features to the current function.

## 1. Introduction

Due to the growing aging population, urination is an essential indicator of health. Lower urinary tract symptoms (LUTSs) are diseases related to the storage of urinary circulation and the excretion phase, including urinary incontinence, night urination, and residual urine. LUTSs are common diseases with a total prevalence rate of 45.2% of the worldwide population (adults over 20 years of age) [1]. LUTSs affect the quality of a patients’ life, and most patients remain unmanaged. Patients have reported lower work productivity, sexuality and overall health levels, and higher rates of depression symptoms and erectile dysfunction than ordinary people [2,3,4,5].

Various tests should be carried out to diagnose LUTS accurately. Of these, checking the condition of the bladder is essential. Methods for evaluating the condition of the bladder include measurements using urinary catheterization or ultrasound. Urinary catheterization involves inserting the catheter into the bladder and measuring the residual amount of urine after urination. It is the gold standard for measuring the post-void residual volume [6]. The invasive method can accurately measure the amount of urine. However, the catheterization makes the patient uncomfortable and shameful. Risks of infection and trauma have also been reported [7,8]. The ultrasound method measures the volume of the bladder with pulse-echo techniques. There are various ultrasound imaging systems designed for 2-D and 3-D sonography. The ultrasound imaging systems are non-invasive and reduce urinary catheterization. There are no significant differences in accuracy compared to catheters [9,10,11]. These imaging systems are difficult to objectively identify the function of the bladder in the process of urine storage and discharge and are intended for use by medical staff for urological diagnosis.

Urodynamic studies (UDSs) are tests to evaluate variations in the lower urinary tract by reproducing urinary symptoms. The UDSs most accurately reflect urodynamics; however, they are invasive and have several limitations as a one-time test. Moreover, there is a fatal disadvantage that only patients who can communicate can be tested. UDSs are more necessary for patients who have a spinal injury or cerebral infarct, or a cerebral hemorrhage. In the case of patients who need an evaluation of urinary dynamics, it is necessary to measure the dynamic changes in the volume of the bladder that can reflect the dynamics of the bladder, which can be measured through the development of wearable systems.

Recently, studies on wearable non-invasive measuring equipment have been conducted using near-infrared spectroscopy [12,13], bioimpedance [14,15], and ultrasound [16,17,18,19,20]. Near-infrared spectroscopy and bioimpedance techniques assume that the optical and electrical properties of pelvic organs are constant and that variations in measurements result from changing the amount of urine in the bladder. The techniques are useful in that they allow relatively free placement of devices and continuous monitoring. However, these methods make it difficult to accurately diagnose patients at a level where the optical and electrical properties allow for an indirect bladder volume estimation. The ultrasound method was used by Kristiansen to measure the bladder volume in 17 men and 13 women by developing an ultrasound monitor of a circular pattern [16]. The measurement accuracy was low when measuring women. Van Leuteren and Kuru measured the length of the bladder in children using wearable ultrasound monitors for children [17,18]. The wearable ultrasound monitors have been commercialized to manage bladder fullness of LUTS patients such as SENS-U kids and Dfree [18,19,20]. The commercial products help manage the users’ urination cycle from the measured bladder length. They are not intended to measure the bladder volume. Therefore, an alternative to UDS requires a continuous bladder volume monitoring system for diagnosing patients.

In this study, we present a prototype of a wearable ultrasound device that can continuously measure the bladder volume during the everyday life of patients who need UDS. This work demonstrates the feasibility of bladder monitoring in wearable ultrasound transducer arrays.

The remainder of this paper is organized as follows. Section 2 presents an approach for the device design. Section 3 describes an acoustic analysis of the design, fabrication process, and volume estimation method. Section 4 presents the implementation results of the proposed methods. Finally, Section 5 and Section 6 present the discussions, conclusions, and avenues for future work.

## 2. Design

### 2.1. Wearable Bladder Scanner Design

**Figure 1:**
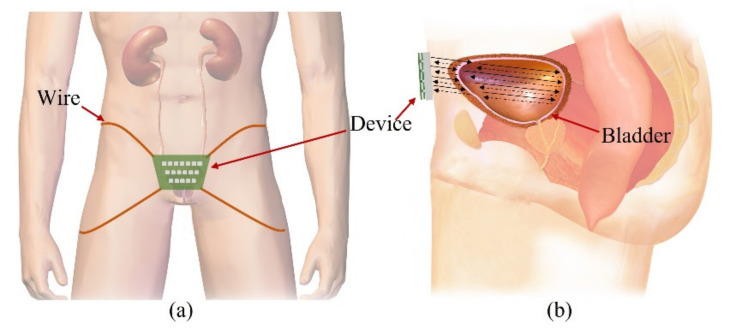
Illustration of the proposed forward-looking wearable urinary bladder scanner system: (**a**) front view and (**b**) side view. This work, “Illustration of the proposed forward-looking wearable urinary bladder scanner system” is licensed under CC BY-SA by Bruce Blaus.

Our goal is to fabricate a wearable device that consists of transducers in a 2-D matrix and constantly monitors the bladder, as shown in Figure 1a. People urinate in the range of 1–2 L a day and urinate in the range of 250–350 mL a time, 4–6 times a day. The normal bladder has a maximum volume of 500 mL. One feels the urge to urinate when the bladder volume is 200–300 mL. The bladder is located in the center of the lower abdomen, close to the pelvic bone. The anterior bladder wall is approximately 40 mm away from the abdominal wall. The full bladder is closer to the abdominal wall than the empty bladder. Depending on the amount of urine, the bladder wall thickness varies from 3 to 15 mm. 

The wearable bladder scanner system is designed as an ultrasound transducer array to measure the position of the bladder wall. It is placed 2 cm above the pubic bone in line with the navel and is secured with wires or belts. The proposed sensing system is based on a forward-looking pulse-echo technique which uses low-profile ultrasound transducers on the lower abdomen. Each element receives reflected signals from the anterior and posterior walls of the bladder, as shown in Figure 1b. Bladder depth is defined as the difference between thresholds of amplitude received at the bladder walls. The volume is estimated by interpolating the shape of the bladder based on the locations of the measured bladder wall.

The bladder can be potentially expanded to more than 8 cm in width and height. The location and curvature vary depending on the size of the bladder. Therefore, the bladder is assumed to be an ellipsoid in this study. The interpolation of features based on location and curvature allows the bladder volume to be estimated by a small number of 2-D arrays, and so it was designed as a 2-D matrix of 5 × 5 channels. The normal size of the bladder after urination is not more than 50 mL and has a width and height of approximately 4 cm. All channels of the transducer array consisted of a pitch of 10 mm and 40 mm × 40 mm to transmit toward the wall of the small bladder after urination.

A piezoelectric transducer with a resonant frequency of 2.2 MHz was used, taking into account the bladder wall thickness and the attenuation of the soft tissue (0.54 dB/cm MHz) [21]. In order to determine the lateral resolution, i.e., the curvature of the bladder, the beam pattern between each element must be separated. The appropriate size of the piezoelectric transducer is determined by considering the limited size of the piezoelectric transducer array and directivity. Directivity is given by

(1) $$\theta =\sin^{-1}\left(1.22\frac{\lambda }{D}\right)$$

where θ is the directivity (radian), λ is the wavelength (mm), and D is the diameter (mm) of the piezoelectric transducer. Piezoelectric transducers have a size of 8 mm × 8 mm with a directivity of less than 10°.

### 2.2. Transducer Design Consideration

Acoustic impedance of piezoelectric material differs significantly from acoustic impedances of body tissues. An impedance mismatch between the body tissues and piezoelectric material causes a low sensitivity and a narrow bandwidth. The performance of devices using piezoelectric materials depends on the proper matching of the acoustic impedance. The problem of acoustic mismatch can be solved using a quarter-wavelength matching layer. Transmission through the matching layer from piezoelectric materials to media is the sum of multiple reverberations within the matching layer. The quarter-wavelength matching layer ensures that all transmission reverberations have the same phase because the wavelength of the piezoelectric material vibrates by half its thickness [22]. When composed of the acoustic impedance of piezoelectric material (*Z_p_*), matching layer (*Z_m_*), and soft tissue (*Z_s_*), the ratio of the transmitted energy to the incident energy on the matching layer is given by

(2) $$E_{t}={\left(\frac{T_{p}T_{s}}{1-R_{p}R_{s}}\right)}^{2}\frac{Z_{s}}{Z_{p}}$$

where *T_p_* = 2*Z_p_*/(*Z_p_* + *Z_s_*) and *T_s_* = 2*Z_m_*/(*Z_m_* + *Z_s_*) are the ratios of the transmitted amplitudes. *R_p_* = (*Z_m_* − *Z_p_*)/(*Z_m_* + *Z_p_*) and *R_s_* = (*Z_s_* − *Z_m_*)/(*Z_s_* + *Z_m_*) are the ratios of the received amplitudes. The acoustic impedance of the matching layer is the geometric mean of the piezoelectric material and soft tissue.

(3) $$Z_{m}=\sqrt{Z_{p}Z_{s}}$$

## 3. Methods

### 3.1. Krimholtz, Leedom, and Matthaei Model Analysis

**Figure 2:**
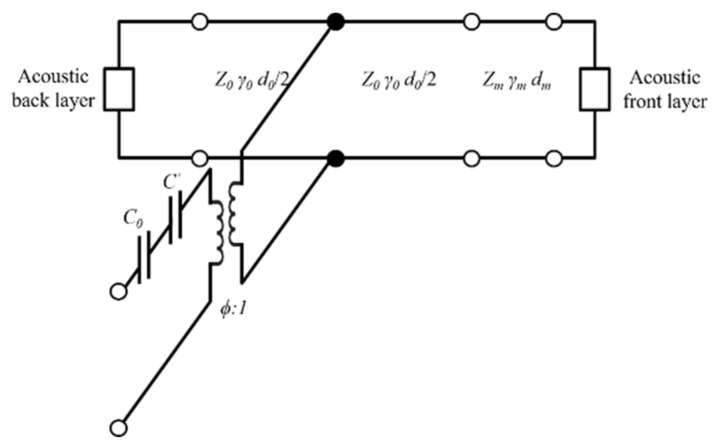
Krimholtz, Leedom, and Matthaei (KLM) equivalent circuit for matching layer.

The Krimholtz, Leedom, and Matthaei (KLM) transmission line model is an equivalent circuit model generally used to determine electrical and mechanical transducer properties [23,24]. The KLM model has the advantage of assuming the acoustic part of the piezoelectric transducer as the transmission line, making it easier to interpret physically and is less time-consuming than finite element analysis (FEA). The frequency response was analyzed using the KLM model to determine the properties of matching layers suitable for soft tissue under ideal conditions. The KLM model consists of a back acoustic layer, piezoelectric material, electric port (*C*_0_, *C’*), matching layer, and a front acoustic layer as shown in Figure 2. It was assumed that the piezoelectric material has air backing layers to maximize the power transfer to the media and to minimize losses. The values of the static capacitance (*C*_0_), capacitance (*C’*), and electrical transformer ratio (*ϕ*) are given by

(4) $$C_{0}=\frac{\varepsilon A_{0}}{d}$$

(5) $$C^{\prime }=-\frac{C_{0}}{k_{t}^{2}\sin\left(\frac{f}{f_{0}}\right)}$$

(6) $$\phi =\frac{k_{t}}{\sqrt{2f_{0}C_{0}Z_{c}}}\sin c\left(\frac{f}{2f_{0}}\right)$$

where *d*, *A*_0_, *Z_c_*, *k_t_*, *ε*, and *f*_0_ are the thickness, area, acoustic impedance of the transducer, piezoelectric coupling constant, permittivity, and resonance frequency, respectively. 

The KLM model is further simplified into a transmission matrix (ABCD matrix) between the electrical and forward acoustic ports [25]. The transmission line model of the piezoelectric transducer or matching layer is defined as a matrix with acoustic impedance, propagation constant, and thickness variables. The propagation constant (*γ*) can be calculated based on the speed of sound (*c*) and the quality factor (*Q*). The ABCD matrix can be expressed as:

(7) $$\gamma =j\frac{2\pi }{c}\left(1-\frac{j}{2Q}\right)$$

(8) $$\left[\begin{array}{cc} A_{i} & B_{i}\\ C_{i} & D_{i} \end{array}\right]=\left[\begin{array}{cc} \cosh\left(\gamma_{i}d_{i}\right) & Z_{i}\sinh\left(\gamma_{i}d_{i}\right)\\ Z_{i}\sinh\left(\gamma_{i}d_{i}\right) & \cosh\left(\gamma_{i}d_{i}\right) \end{array}\right]$$

where *i* can be expressed as a transmission line matrix of the piezoelectric transducer layers, matching layers, and acoustic layers. The relationship between the transmission matrix with the circuit and piezoelectric transducer is as follows:

(9) $$\left[\begin{array}{c} F\\ u \end{array}\right]=\left[\begin{array}{cc} A & B\\ C & D \end{array}\right]\left[\begin{array}{c} V\\ I \end{array}\right]$$

where *V* is the source voltage, *I* is the source current, *F* is the force on the front face, and *u* is the particle velocity at the front face. The ABCD matrix coefficient is obtained by multiplying the individual matrix corresponding to the electrical matrix, electromechanical matrix, piezoelectric transducer, and matching layer. If the source impedance (*Z_s_*) and voltage are known, the transmission sensitivities can be computed as

(10) $$P=\frac{F}{VA_{0}}=\frac{Z_{a}}{\left\{Z_{s}\left(CZ_{a}+D\right)+AZ_{a}+B\right\}A_{0}}$$

Based on previous considerations, the KLM model was used to determine the properties of the matching layer, and the frequency response characteristics were evaluated.

### 3.2. Finite Element Model Analysis

**Figure 3:**
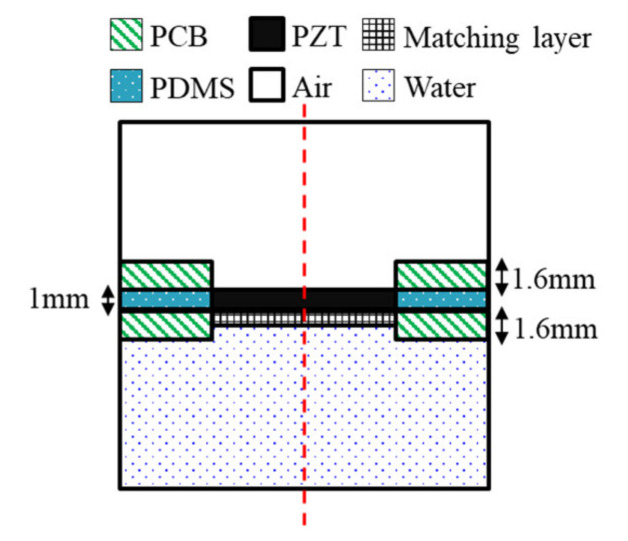
Geometry of the finite element model.

FEA was conducted using commercial FEA package simulation software (COMSOL Multiphysics, COMSOL Inc., Stockholm, Sweden) to predict the distribution of the pressure and sound pressure by the frequency of the device being designed. The FEA was designed based on the frequency determined from the design of the wearable bladder scanner. The FEA was made of 2-D axial symmetry plane elements to reduce the size of reconstruction problems and calculation time. A geometry was modeled with an integrated plate and piezoelectric transducers, as shown in Figure 3. The total size of the model was 35 mm width and 95 mm height. The geometry and material properties of the model are based on a fabricated piezoelectric element. The following boundary conditions were applied: the entire structure was free. The piezoelectric transducer, plate, and matching layer consisted of elastic layers. A potential of 1 V was applied to the piezoelectric transducer’s top surface, and the bottom surface of the piezoelectric transducer contacting the matching layer was grounded. The mesh size of each material consisted of rectangular elements less than 1/10 of the wavelength at the frequency of interest. All external boundaries of the calculated area were defined as absorption boundary conditions.

The piezoelectric material selected was PZT-5A, 8 mm in length and 1 mm in thickness. The plate was 1.6 mm thick, and the FR-4 material was used in the printed circuit board (PCB). A matching layer material should have an acoustic impedance of 7.6 MRayl to improve coupling with PZT-5A (35.42 MRay) and soft tissue (1.63 MRayl) from Equation (3). The matching layer was a mixture of epoxy and aluminum oxide powders [26]. The acoustic impedance was 7.62 MRayl, and the thickness was 373 μm, which is a quarter wavelength. The space between the plates was filled with polydimethylsiloxane (PDMS). In order to improve the power to the front medium, an air-backed transducer configuration was used. A hole was created on the back of the piezoelectric transducer for air backing, and the hole had a diameter of 7.6 mm. The front medium was set with water similar to the acoustic impedance of the soft tissue. The physical parameters of the materials used in the FEA are listed in Table 1. The simulations were conducted with the presence or absence of matching layers under piezoelectric transducers. The frequency response signals were extracted at a distance of 60 mm from the piezoelectric transducer. 

### 3.3. Fabrication of the Wearable Bladder Scanner

**Figure 4:**
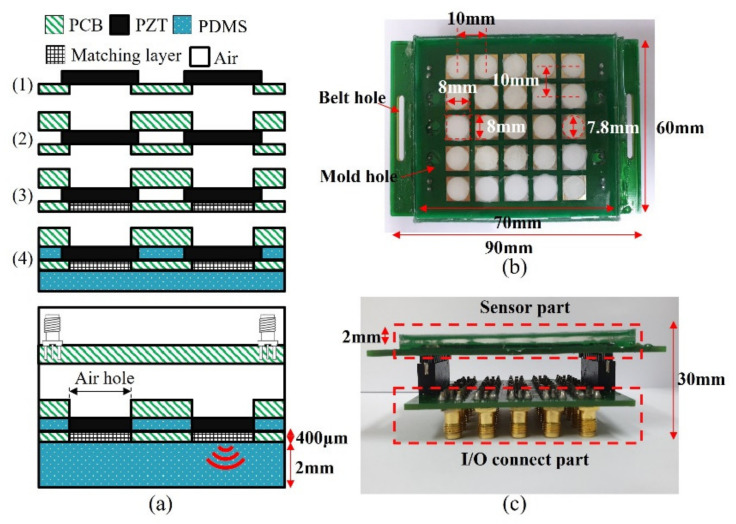
(**a**) Manufacturing process prototype of proposed wearable bladder scanner. Photograph of fabricated device: (**b**) bottom view and (**c**) side view combined with an I/O connection part.

Ultrasound transducers in the wearable bladder scanner were implemented as an array configured with 1 mm thick PZT-5A (T180-A4N0-2929, Piezo.com, Woburn, MA, USA). Instead of PZT-5H with excellent output performance, PZT-5A is useful for other processes because it is not sensitive to temperature changes. PZT-5A was cut into sizes of 8 mm × 8 mm using a laser dicing, considering its directivity and size. The piezoelectric transducers consisted of a 5 × 5 array with a pitch of 10 mm per element. Two plates were integrated with the piezoelectric array. The plate between the piezoelectric element and the medium of interest was manufactured with a 400 μm thick plate to adjust the thickness of the matching layer. The material of the plates was PCB (FR-4) for electrical connections. A prototype of the wearable bladder scanner was produced using the following production process as shown in Figure 4a. Piezoelectric transducers were bonded with the plates using a conductive epoxy (CW 2400, Chemtronics Inc., Kennesaw, GA, USA). Each piezoelectric transducer was electrically connected to the plates. The conductive epoxy consisting of two parts should be mixed for 1 min at a ratio of 1:1. A hot plate can be used to cure conductive epoxy quickly. The matching layer was produced using 200 nm oxide aluminum powder (AV-AL803200N, Avention, Incheon, Korea) and epoxy (EPO-TEK 301, Epoxy Technology Inc., Billerica, MA, USA) to match the acoustic impedance of the piezoelectric transducer and soft tissue. EPO-TEK 301 consisted of resin and a hardener, mixed using a 4:1 weight ratio, and was stirred. After the two parts were shaken sufficiently, EPO-TEK 301 was mixed with aluminum oxide powder in a 1:1.3 weight ratio and was stirred for 10 min. The mixture was degassed in a pressure chamber using a vacuum pump (15,500, Robinair Inc., Warren, MI, USA). The inside of the pressure chamber was maintained at 100 kPa for 20 min. The mixture was applied to the piezoelectric transducer at 400 μm, about the quarter wavelength, and cured for 24 h at 25 °C. The inside and front of the device were molded with PDMS to prevent electrical interference and contamination between piezoelectric transducers. PDMS was degassed in the same way and heat was cured at 80 °C for 2 h.

In this prototype, a total of 25 piezoelectric transducers were used and distributed on a rectangular surface of 7 cm × 6 cm as shown in Figure 4b. The operation of the transducer can be controlled by connecting the cable to the I/O connection part (Figure 4c). In clinical practice, more transducer elements or other plate materials may be required to estimate the bladder volume.

### 3.4. Volume Estimation Method

The shape of the urinary bladder is typically assumed to be an ellipsoid, triangular prism, cube, or cylinder to estimate the volume [27]. The bladder volume is calculated by multiplying the width, depth, height, and the specified coefficient for each shape. Each dimension is the maximum diameter derived from the transverse and longitudinal views using 2-D ultrasound imaging equipment. The volume estimation method of the bladder is mainly recommended as an ellipsoid because of its relatively accurate results and ease of application [28,29]. Among the shapes assumed as the bladder, the ellipsoid can be derived by a single quadric surface equation. The ellipsoid cylinder was excluded due to maximum height inaccuracy. The quadric surface is a general equation of three coordinates (x, y, z) and nine parameters. The nine parameters can represent invariants consisting of coefficients of the general equation that do not change in parallel transformation or rotation [30]. The invariants can be assumed as constraints represented by an ellipsoid. The parameters can quickly fit the ellipsoid to the data using least-squares optimization. The optimization can produce other shapes, such as hyperboloids or paraboloids. To prevent this, we used the constraints of linear combinations of two terms known as invariants presented by Li and Griffiths [31]. 

To obtain the parameters of the equation requires more data than the number of parameters. The origin is set to the center of the transducer array. The orthogonal direction toward the bladder is set to the *z*-axis. The fabricated system provides up to 50 points of the bladder wall coordinates. On the side of the bladder, the distinction of the bladder walls is unclear, and the data contains noise. The *x*-axis and *y*-axis data intersecting the bladder center are fitted to the ellipse to minimize the effect of noise on the side of the bladder. The points in the measured data are weighted according to the peak size. The number of weighted points is similar to the number of points in the ellipse intersecting the bladder center. The bladder volume is estimated from the diameters of the fitted ellipsoid.

### 3.5. Experimental Setup

**Figure 5:**
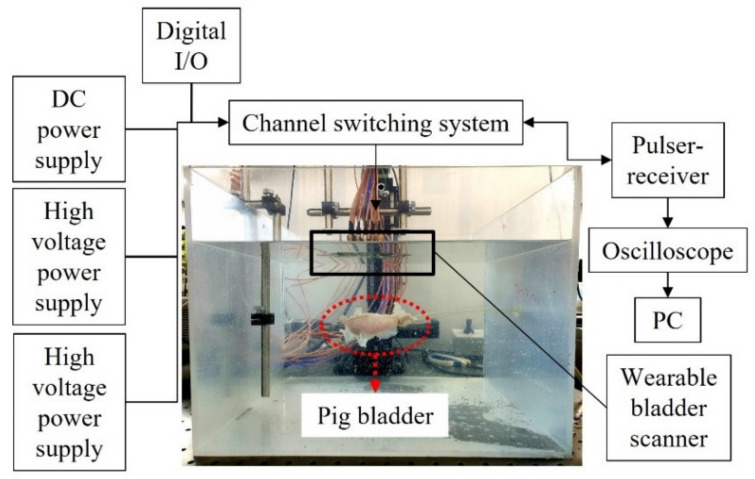
Experimental setup for the measurement of pig bladder wall position.

The feasibility test of the wearable bladder scanner prototype was performed on a pig bladder in a water tank (Figure 5). The pig bladder was obtained through a slaughterhouse and no additional harm was applied to any animal. In the preparation, air bubbles inside the bladder were removed and the bladder was connected to a silicon tube. The pig bladder was injected with 450 mL of water in 50 mL increments to assume the filling process of the bladder. The anterior wall of the bladder was placed 100 mm perpendicular to the axis of the device. A pulser-receiver system (5072PR, Olympus Inc., Tokyo, Japan) transmitted signals to the transducers in the −145 V spike pulse and was conditioned with a 40 dB gain and 1 MHz high-pass filter to filter the ultrasound echoes. A switch (CPC7601, IXYS Corporation, CA, USA) was used to control multiple channels and select the transmission and reception channels. The operating voltages of the switch were 140 and −60 V, resulting in a potential difference of 200 V for switching. A digital I/O device (NI USB-6501, National Instruments Corp., Austin,, TX, USA) was used to apply logical voltages for channel switching. The received acoustic signal was obtained using an oscilloscope (DSO1014A, Keysight Technologies, Santa Rosa, CA, USA) with a sampling frequency of 2 GHz. The switch controlled by a written program acquired one channel of data and then changed to another channel for the next data. All data channels were acquired for a total of 60 s. Back-end processing of bladder volumes was performed with the 25 scan lines.

## 4. Results

### 4.1. Acoustic Analysis Results

**Figure 6:**
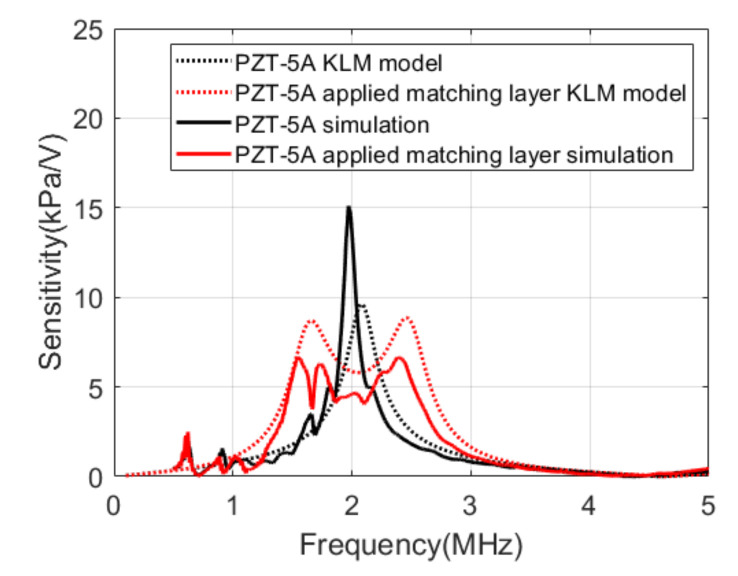
Transmit sensitivities of the Krimholtz, Leedom, and Matthaei (KLM) models and the simulations.

Figure 6 compares the results of the KLM model analysis with the results of the simulations. The center frequency of PZT-5A was 2.1 MHz, and the bandwidth was approximately 11% in the KLM model. The simulation results of integrating PZT-5A and PCB represented a center frequency of 2 MHz and a bandwidth of 5%. When the matching layer was applied, the sensitivity decreased by 4.6 dB, and the bandwidth increased to 51.8% in the KLM model. In the simulation, the sensitivity decreased by 10.3 dB, and the bandwidth increased to 53.6%.

### 4.2. Performance Evaluation of the Fabricated Device

**Figure 7:**
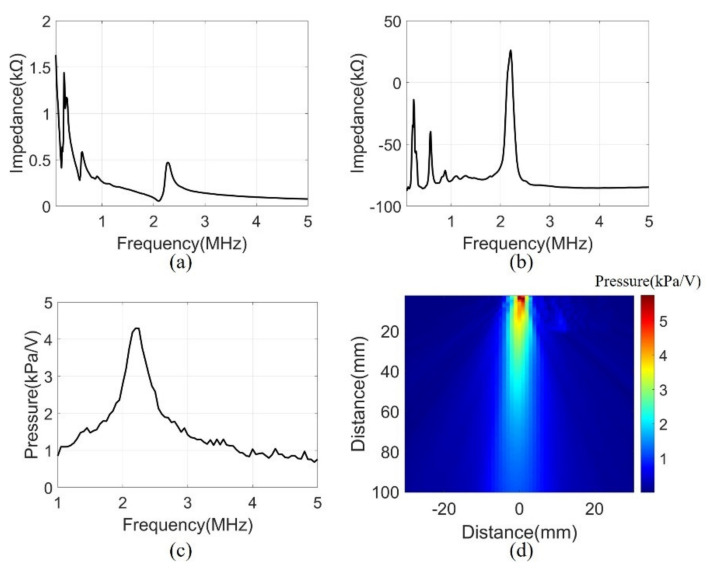
(**a**) Electrical impedance magnitude, (**b**) Electrical impedance phase, (**c**) Transmit sensitivity at 100 mm, (**d**) Pressure field of 2.2 MHz in water.

A series of measurements were conducted to assess the performance of the manufactured system. The electrical input impedance was measured in the air using an impedance analyzer (4194A, Agilent Technologies, Inc., Philadelphia, PA, USA). The measurement was performed in the frequency range of 0.1 to 5 MHz. Figure 7a,b show the magnitude and phase of the electrical impedance, respectively. At the resonant frequency of 2.2 MHz, the electrical impedance (208 Ω) and the phase (26°) showed the mechanical vibration of the device. Several peaks at a low frequency were due to the mode of vibration in the lateral direction.

Transmit sensitivity was measured to evaluate the bandwidth of the array elements with the matching layer. The measurement was performed underwater using a hydrophone (HNR0500, ONDA, Sunnyvale, CA, USA) at the height of 100 mm from the array element. A pulse generator (33500B, Keysight Technologies, Santa Rosa, CA, USA) was used to generate signals of AC 10 V and 10 cycle bursts. The frequency range was 1-5 MHz and were measured in 50 kHz steps. The data measured by the hydrophone were calibrated using the reception sensitivity of the datasheet. The received data had a center frequency of 2.2 MHz and a −3 dB bandwidth of 16% (Figure 7c).

The acoustic pressure field was measured to evaluate the sound pressure levels and angle of directivity of the beam propagation. The measurement scanned the range of 60 mm × 100 mm in 1 mm units using a motorized control stage (SM3-0820-4S, Sciencetown Inc., Incheon, Korea). The transducer was excited at a 2.2 MHz frequency by the pulse generator. The signal was received underwater by a hydrophone and was calibrated to reception sensitivity. Figure 7d shows the pressure field derived from the maximum value of the measurement data. The surface acoustic pressure was 6 kPa/V, and the calculated angle of −3 dB directivity was 9.56°.

### 4.3. Volume Estimation Experimental Results

**Figure 8:**
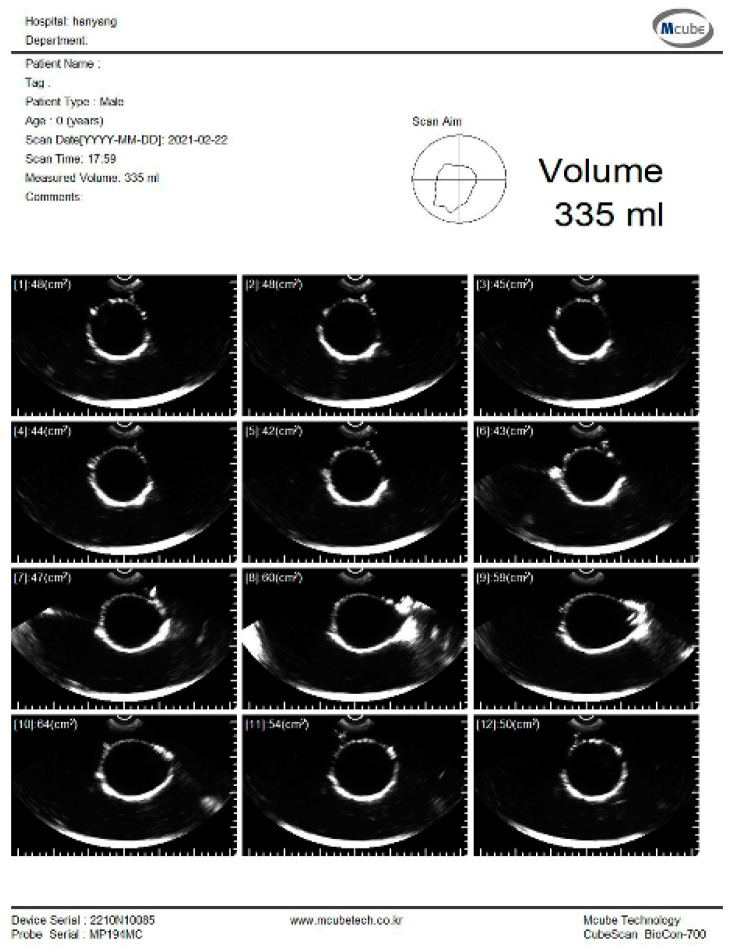
B-mode images and estimated volume value of the pig bladder filled with 300 mL of water.

The experiment was conducted to estimate the volume using the pig bladder to verify the feasibility of the system. Whenever 50 mL of water was injected, the bladder volume was measured using a commercial bladder scanner (BioCon-700, MCUBETCH, Seoul, Korea). The working frequency of BioCon-700 is 2.6 MHz. Based on the manufacturer’s specification, the accuracy of volume estimation is ± 15%, when the target volume is greater than 100 mL (or ±15 mL on volumes less than 100 mL). Immediately after the commercial equipment measurement, 25 channel data were obtained using the fabricated device. The commercial equipment provided B-mode images and a volume estimation value of the pig bladder as shown in Figure 8. All measurements, which are performed with the commercial equipment, are in the reference images as shown in subfigures (a) in Figures S1–S7.

**Figure 9:**
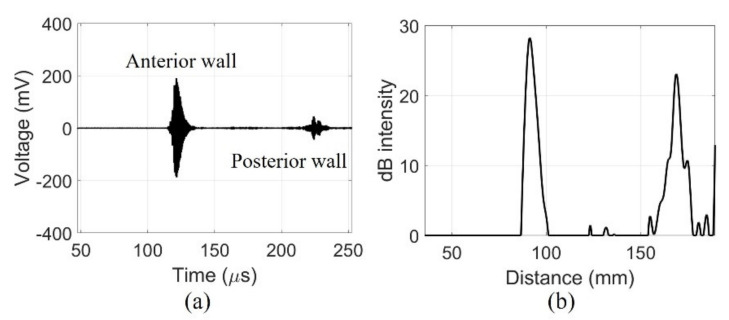
Echo signal received by a single element of fabricated device centrally in the 300 mL of water-injected pig bladder: (**a**) filtered data and (**b**) dB scale.

The fabricated device detected signals from the walls of the pig bladder filled with water. The echo received from the center of the pig bladder can distinguish the bladder wall. The amplified signals obtained a clear signal with a center frequency of 2.2 MHz using a band-pass filter of 1.1–4.4 MHz as shown in Figure 9a. The dimensions of the bladder can be determined by distinguishing the position of the bladder wall with the signals generated by the impedance mismatch. The received signal from the posterior wall of the bladder was amplified to compensate for the diffraction loss before filtering. In the case of in-vivo measurements, additional time gain compensation should be required due to the attenuation of tissue. The compensated and filtered data were processed by an envelope detection and was later converted to dB scale to position the measured bladder wall as shown in Figure 9b. The maximum depth of the bladder measured by the commercial equipment was 75 mm. The distance between the peaks of the fabricated device was 77.6 mm. The bladder depth indicates that the measurements of the two devices are similar. 

**Figure 10:**
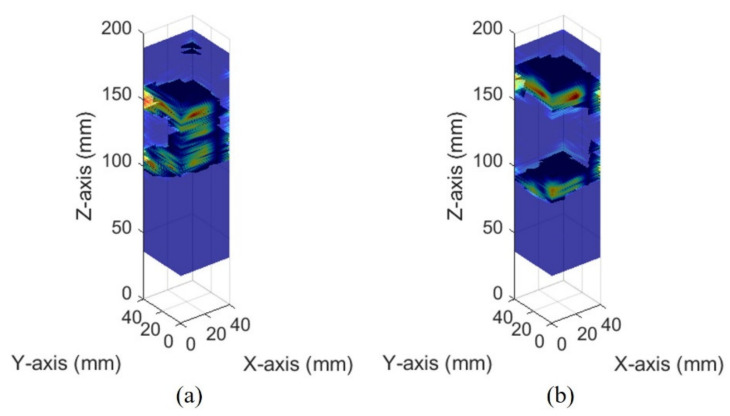
Ultrasound pulse-echo 3-D images of the pig bladder walls: (**a**) 50 mL and (**b**) 300 mL.

**Figure 11:**
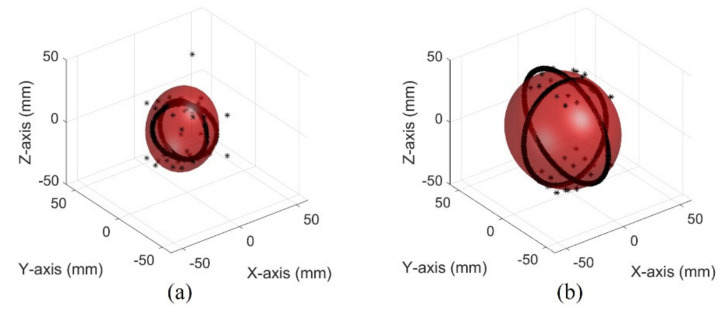
Optimized ellipsoid from measured data in the pig bladder: (**a**) 50 mL (**b**) 300 mL.

The images of different bladder volumes based on dB scale data show the shape of the bladder (Figure 10). The field of view of the fabricated device did not cover the entire bladder, resulting in a part of the bladder that can be seen. The bladder parts showed that the position and curvature of the anterior and posterior walls of the bladder varied depending on the bladder volume. Coordinates of the bladder walls were extracted from the dB scale data of each element. The bladder coordinates were used as the first peaks of the bladder wall signals. The second echo signal of the anterior bladder wall was excluded as an error factor for estimating the volume during the extraction process. The parameters of the quadric surface were optimized with the extracted points to fit into an ellipsoid. Two examples are presented in Figure 11. The radii of the fitted ellipsoid were calculated using the eigenvectors. All optimized ellipsoid images are in the reference image, as shown in subfigures (b) in Figures S1–S7.

**Figure 12:**
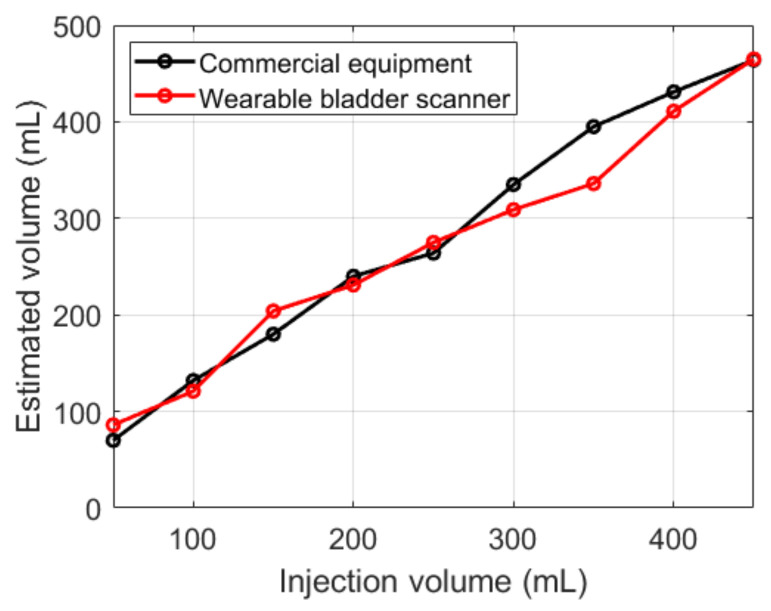
Estimated bladder volumes with various injection volumes.

Both devices showed an overall overestimation of the measured volume relative to the injection volumes (Figure 12). The results of the fabricated device were similar to those of the commercial equipment, except for 350 mL. Compared with the injection volumes, the commercial equipment showed an average absolute error of 29 mL, and the fabricated device showed 24 mL.

## 5. Discussion

Obtaining ongoing information about urine production and excretion is vital for future clinical research. Therefore, we designed a new wearable ultrasound bladder scanner for continuous monitoring. The prototype device is intended for various urological patients and is focused on patients who need UDS.

We reduced the effect on propagation owing to the high difference between the transducer and the soft tissue acoustic impedance by attaching matching layers. The system integrated with PCBs was consistent with the pressures shown in the acoustic analysis. However, the bandwidth observed by acoustic pressure evaluation was not impressive compared with the acoustic analysis results. Using several matching layers and/or a backing layer could further increase the bandwidth and resolution.

We presented a method for estimating the volume of the bladder volume with a small number of elements. The bladder volume was assumed to be an ellipsoid derived as a formula with a quadric surface. The proposed volumetric method resulted in a similar accuracy compared to the commercial bladder scanner. The commercial bladder scanner was shown to be outside the accuracy specification at a volume of 200 mL or less in ex-vivo measurements. In the case of in-vivo measurements, the accuracy of the commercial scanner may be improved. The estimated volumes of both the commercial bladder scanner and the proposed volumetric system presented a linear relationship with the injected volume. Clinical trials using the developed forward-looking sensor are required to determine the volume accuracy of urinary bladders with various volumes.

The proposed method was validated in the water. For clinical application, several factors should be considered such as attenuation, acoustic impedance, and scattering. The human body interior reduces the amplitude of the ultrasound signal with attenuation depending on the depth. The attenuation factor is approximately 1.2 dB/cm at 2.2 MHz [21]. Adjusting the parameters of time gain compensation can balance the attenuation in abdominal tissue. The acoustic impedance of soft tissue is different from that of water; however, the acoustic impedance of soft tissue is similar to that of water compared to a piezoelectric element with a high acoustic impedance. Scattering occurs when ultrasound is propagating through nonhomogeneous media, such as internal organs [22]. As a result, typical tissue imaging has speckle regions. In this study, the location of the bladder wall is defined as the first peak of the bladder wall signals. Distinguishing bladder wall signals can be difficult due to the speckle region in in-vivo measurements. Since urine in the bladder does not generate scattering, the detection method of the bladder walls can be improved based on the speckle region around the bladder.

The proposed method for estimating the bladder volume has the advantage of being faster than the method for measuring the volume using ultrasound images. In addition, the volume can be estimated without the user detecting the optimal bladder location. Volumetric methods using ultrasound images are measured using mechanical scanning. This method is time-consuming from mechanical limitations. The ultrasound imaging systems are challenging to fabricate as wearable devices and are cost-ineffective due to their scanning shape.

## 6. Conclusions

This study presents a wearable bladder scanner for continuous volume monitoring. The bladder scanner was implemented as a system with a 5 × 5 piezoelectric elements array integrated on thin substrates. The system had a 2.2 MHz central frequency and 16% bandwidth. Bladder volume was estimated by optimizing an ellipsoid using the quadric surface equation. The inserted data in the optimization were extracted using the first peaks of the anterior and posterior bladder wall signal as location information. With the injection volume ranging from 50 mL to 450 mL, the proposed system and commercial equipment had an average error of 24 mL and 29 mL, respectively. The experiment demonstrated the feasibility of bladder monitoring using a 2-D ultrasound transducer array system relative to commercial equipment.

In future work, clinical trials using the developed forward-looking sensors are planned to determine the volume accuracy of urinary bladders. In addition, configuration of flexible substrates and expansion of the current volume estimate method are essential for different abdominal curvatures for each individual. It is clear that a flexible 2-D array system will contribute to the development of electronic voiding diaries as part of the home healthcare system.

## Figures and Tables

**Table 1:** Material properties used for modeling.

Property	PZT-5A	Matching Layer	FR-4	PDMS
Density (kg/m^3^)	7700	2295	1850	1000
Speed of sound (m/s)	4600	3320	3602	1500
Acoustic impedance (MRayl)	35.42	7.62	6.66	1.5

## Data Availability

Not applicable.

## Supplementary Materials

The following are available online at https://www.mdpi.com/article/10.3390/s21165445/s1, Figure S1: Images of pig bladder injected 100 mL of water (a) commercial equipment and (b) optimized ellipsoid from the measured data. Figure S2: Images of pig bladder injected 150 mL of water (a) commercial equipment and (b) optimized ellipsoid from the measured data. Figure S3: Images of pig bladder injected 200 mL of water (a) commercial equipment and (b) optimized ellipsoid from the measured data. Figure S4. Images of pig bladder injected 250 mL of water (a) commercial equipment and (b) Optimized ellipsoid from the measured data. Figure S5: Images of pig bladder injected 350 mL of water (a) commercial equipment and (b) optimized ellipsoid from the measured data. Figure S6: Images of pig bladder injected 400 mL of water (a) commercial equipment and (b) optimized ellipsoid from the measured data. Figure S7: Images of pig bladder injected 400 mL of water (a) commercial equipment and (b) optimized ellipsoid from the measured data.
