# Supplementary material for: Forward-Looking Ultrasound Wearable Scanner System for Estimation of Urinary Bladder Volume
Source: Sensors (Basel). 2021 Aug 12;21(16):5445. doi: 10.3390/s21165445 (PMC8400094; doi:10.3390/s21165445)
Supplement: Supplementary file 1 [file sensors-21-05445-s001.zip › sensors-1320884-supplementary.pdf]

sensors

MDPI

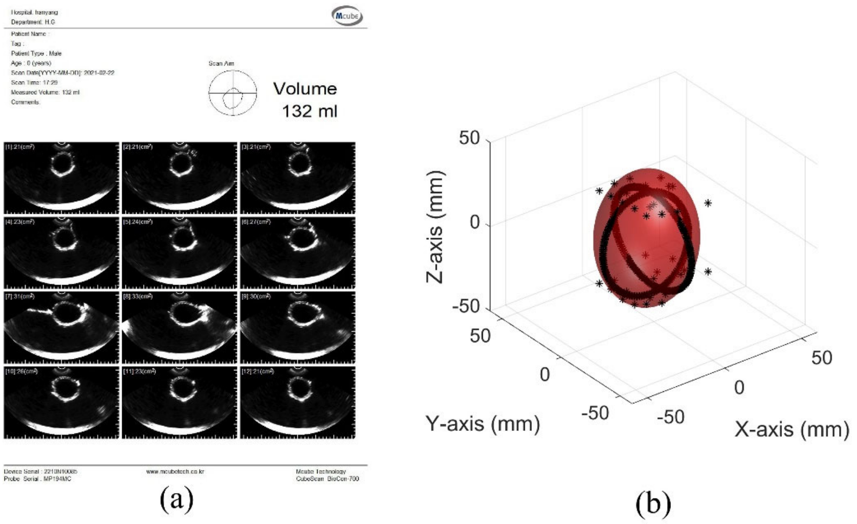

Figure S1. Images of pig bladder injected 100 mL of water (a) commercial equipment and (b) optimized ellipsoid from the measured data.

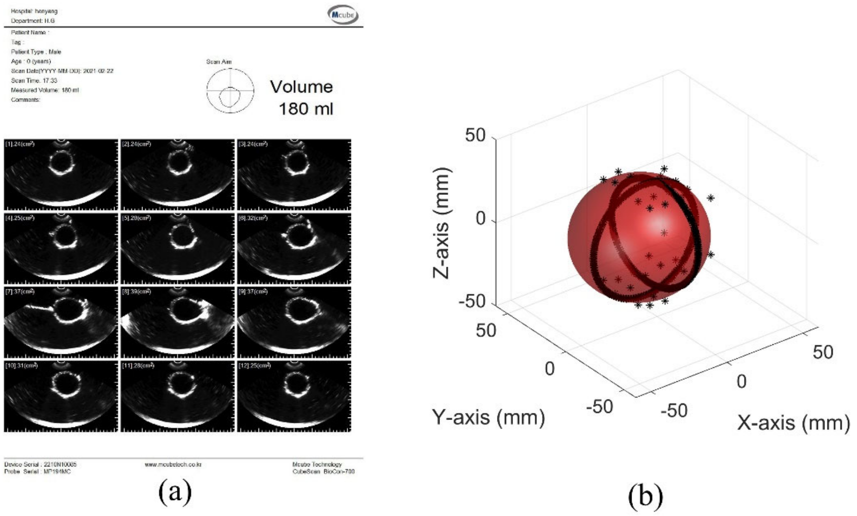

Figure S2. Images of pig bladder injected 150 mL of water (a) commercial equipment and (b) optimized ellipsoid from the measured data.

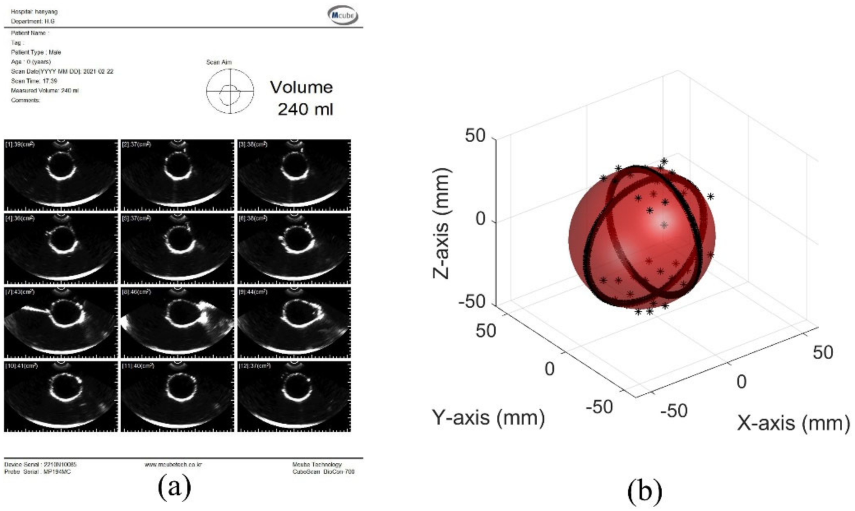

Figure S3. Images of pig bladder injected 200 mL of water (**a**) commercial equipment and (**b**) optimized ellipsoid from the measured data.

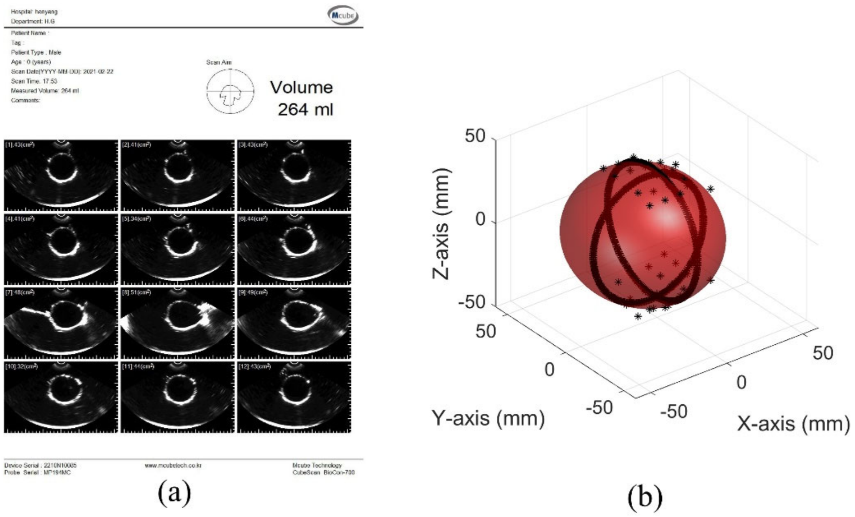

Figure S4. Images of pig bladder injected 250 mL of water (**a**) commercial equipment and (**b**) optimized ellipsoid from the measured data.

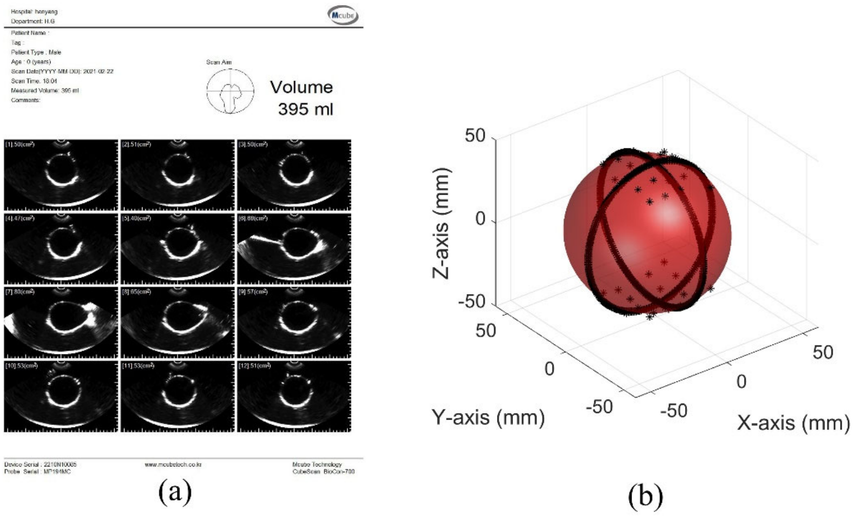

Figure S5. Images of pig bladder injected 350 mL of water (**a**) commercial equipment and (**b**) optimized ellipsoid from the measured data.

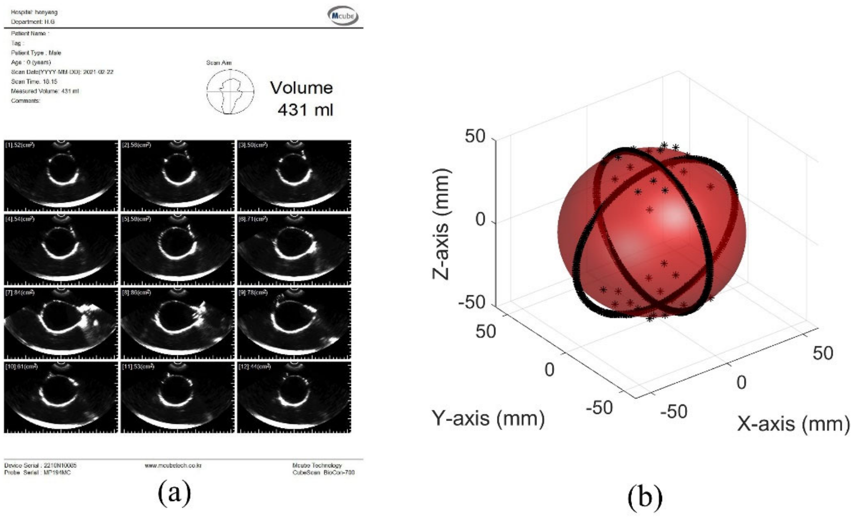

Figure S6. Images of pig bladder injected 400 mL of water (**a**) commercial equipment and (**b**) optimized ellipsoid from the measured data.

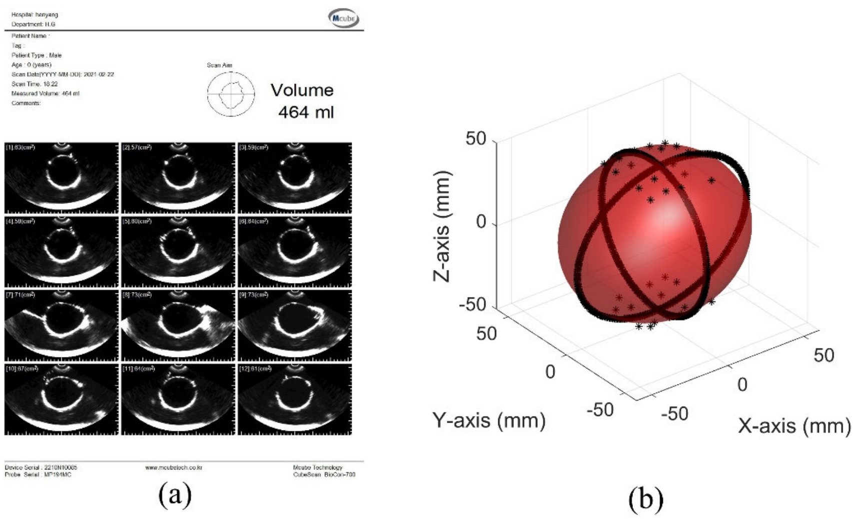

Figure S7. Images of pig bladder injected 400 mL of water (**a**) commercial equipment and (**b**) optimized ellipsoid from the measured data.
